# Confirmatory factor analysis and gender invariance of the Persian version of psychological control scale: association with internalizing and externalizing behavior problems

**DOI:** 10.3389/fpsyg.2023.1128264

**Published:** 2023-12-14

**Authors:** Mojtaba Habibi Asgarabad, Pardis Salehi Yegaei, Sima Mokhtari, Balal Izalnoo, Elizabeth Trejos-Castillo

**Affiliations:** ^1^Health Promotion Research Center, Iran University of Medical Sciences, Tehran, Iran; ^2^Department of Psychology, Norwegian University of Science and Technology, Trondheim, Norway; ^3^Department of Health Psychology, School of Behavioral Sciences and Mental Health (Tehran Psychiatric Institute), Iran University of Medical Sciences, Tehran, Iran; ^4^Positive Youth Development Lab, Human Development and Family Sciences, Texas Tech University, Lubbock, TX, United States; ^5^Center of Excellence in Cognitive Neuropsychology, Institute for Cognitive and Brain Sciences, Shahid Beheshti University, Tehran, Iran; ^6^Department of Clinical Psychology, School of Behavioral Sciences and Mental Health (Tehran Psychiatric Institute), Iran University of Medical Sciences, Tehran, Iran; ^7^Faculty of Psychology, Kharazmi University, Tehran, Iran; ^8^Human Development and Family Sciences, Texas Tech University, Lubbock, TX, United States

**Keywords:** internalizing and externalizing behavior problems, gender invariance, parental psychological control, reliability, validity

## Abstract

**Introduction:**

The current cross-sectional study aimed to examine the reliability, construct validity, gender invariance and concurrent validity of the psychological control scale-youth self-report (PCS-YSR) among Iranian adolescents.

**Methods:**

A total of 1,453 high school students (49.2% boys; M_*age*_ = 15.48, SD = 0.97), who aged between 14 and 18 years old completed the PCS-YSR and the youth self-report (YSR) scale of behavior problems.

**Results:**

Reliability was established using Cronbach’s alpha and ordinal alpha for maternal and paternal psychological control. The confirmatory factor analysis (CFA) results supported the original unidimensional model of the PCS-YSR scale for both mother and father forms. Results also revealed that mother and father forms of PCS-YSR were invariant across adolescents’ gender. When comparing the mean differences, mothers were more psychologically controlling toward their sons, compared to their daughters. The mother and father forms of PCS-YSR were found to have acceptable concurrent validity through their relationship to internalizing and externalizing behavioral problems.

**Discussion:**

Overall, our findings supported the psychometric properties of the Persian version of the Psychological Control Scale-Youth Self-Report among Iranian adolescents. This scale can be used as an efficient tool for parental psychological control among adolescents in Iran. The negative effect of the intrusive parenting behavior on child’ negative outcomes in Iran, irrespective of culture, was shown.

## Introduction

During adolescence, the successful development of an autonomous stand toward parents is highly salient. Though parents might be expected to authenticate this independent stance that would support healthy future relationships for their children (Oudekerk et al., 2015), some parents attempt to maintain their influence on their child’s psychological world through intrusive strategies of psychological control. Schaefer (1965a) first coined the term psychological control and argued that controlling parents refuse to accept the children’s autonomy, which inevitably interfere with adolescent development (Schaefer, 1965b). To elaborate, Barber (1996) defined psychological control as parenting behaviors with forcible intrusion into children’s opinions, values, and emotions. Steinberg (1990) and Barber (1996) also posited the existence of four psychological control strategies, namely, guilt-induction, anxiety infusion, love withdrawal, and invalidating the child viewpoint. Parental psychological control, which could have negative effects on adolescents’ mental health (Steinberg, 1990; Romm et al., 2020), needs to be differentiated from a positive parenting style of behavioral control aiming to regulate and shape children’s behavior, such as manners, study activities, and peer social interactions (Dishion and McMahon, 1998; Stattin and Kerr, 2000).

A few instruments assess parental psychological control, such as the subscale of Psychological Control vs. Autonomy from the Children’s Report of Parents’ Behavior Inventory (CRPBI; Schaefer, 1965a), is used to evaluate psychological control. The Parental Behavior Inventory (PBI; Lovejoy et al., 1999) that evaluates aspects such as parental demands for obedience, intrusive behavior, and manipulation of affective responses. However, these scales are not focused only and directly on parental psychological control, but measure it alongside or mixed with other parenting behaviors. As a reflection of his proposed theory, Barber (1996) developed the Psychological Control Scale-Youth Self-Report (PCS-YSR), which is the most widely applied measure of youths’ perception of their parents’ psychological control (Soenens and Vansteenkiste, 2010). This scale assesses the degree that the adolescents appraise the level of psychological control in their parents through their use of strategies like feelings invalidation, suppression of verbal expression, guilt induction, love withdrawal, and unpredictable or unstable emotional behaviors. The PCS-YSR is a self-report questionnaire for youths with two separate forms for mothers and fathers.

Although the 16-item PCS-YSR was originally unidimensional (Barber, 1996), some researchers have investigated the factor structure of this scale, finding that the best fit includes three-factors, namely, invalidating feelings, personal attack, and love withdrawal (Luyckx et al., 2007; García-Pérez et al., 2019; Romm et al., 2020). Previous evidence has supported the satisfactory psychometric properties of this scale across several countries (Barber et al., 2012; Metin-Orta and Metin-Camgöz, 2021). For example, the internal consistency for maternal and paternal control were acceptable (0.62 and 0.67) in an American sample (Barber et al., 2005a), and good (0.83 and 0.76) in the Spanish population (Rodríguez-Menéndez et al., 2021). Regarding the validity of PCS-YSR, the body of research has addressed the negative effect of this parenting behavior on adolescents’ substance use (Romm and Metzger, 2018), depressive symptoms (Bleys et al., 2018; Wang et al., 2022), risky cyber behaviors (Romm et al., 2020), delinquency (Zhu and Shek, 2021), and aggression (Murray et al., 2014; He et al., 2019). Given that manipulative techniques of psychological control are used to inhibit the stabilization of a secure sense of self in adolescents and intrude in their psychological world, controlling parents impose the risk of internalizing problems on adolescents (Barber, 1996). In addition, using intrusive behaviors and guilt induction, parental psychological control leave adolescents prone to insecurity and frustration and consequently, to externalizing problems (De Kemp et al., 2006). In other words, adolescents aggressive behaviors are indications of conflict with negative family atmosphere (Stone et al., 2002). The positive association of PCS-YSR with internalizing behaviors, and to some degree, with externalizing problems has also been documented (Janssens et al., 2015; Kaniušonytė and Žukauskienė, 2016; Lu et al., 2017; Van Heel et al., 2019). For instance, Stone et al. (2013) in their study on 298 children found that psychological control has a positive association with internalizing and externalizing problems (Stone et al., 2013). These results have been repeated in adolescent population. Cui et al. (2014) showed that psychological control has a direct relationship with depressive symptoms and aggressive behaviors in adolescents (Cui et al., 2014).

One of the most important gaps in the field of parental psychological control is the shortage of evidence on distinct paternal and maternal psychological control patterns toward sons and daughters across diverse cultural and national backgrounds. Barber et al. (2005b) and Romm et al. (2020) studies indicated that the overall pattern of psychological control level is comparable, irrespective of child’s and parent’s gender. However, studies addressing gender discrepancies showed inconsistent results, some indicating higher parental psychological control toward boys (Nelson and Crick, 2002; Endendijk et al., 2016), while others found that parents tended to be more controlling with their daughters (Linver et al., 2002; Domènech Rodriguez et al., 2009). Potential gender differences may be explained through the bio-social theory (Wood and Eagly, 2002, 2012). This theory argues that in the context of gender stereotypes, girl and boy adolescents are treated differently by each parent (Wood and Eagly, 2012). Endendijk et al. (2016) in their review study found that from 1990 onward parents behave with more forcefully and authoritative behaviors toward their sons, whereas employ autonomy supportive strategies (such as empathy, kindness, and relational harmony) for their daughters. Additionally, despite rapid shifts from a traditional and collectivistic culture to individualistic and modern values in Iranian society (Abbasi et al., 2002), some gender stereotypes still exist in the parenting behaviors. Therefore, further evidence concentrated specifically on comparing the level of mother and father control independently on boy and girl adolescents is needed. It can shed light on how gender dynamics affect parenting strategies, explore their distinct consequences for adolescent development more comprehensively, provide insights into whether girls and boys manifest behavior problems differently in response to parental control strategies, and lead researchers and practitioners to tailored strategies and more culturally sensitive interventions of parental control. The current study is first-of-its-kind that as a secondary aim, explored the gender differences in mothers’ and fathers’ level of psychological control toward girl and boy adolescents in Iran, as a Middle-eastern country.

To address the abovementioned gaps in the literature, the present study aimed to evaluate the psychometric features of the PCS-YSR among Iranian adolescents. To elaborate, we aimed to examine: (a) *Cronbach*’s alpha, composite reliability, and Ordinal alpha reliability tests of PCS-YSR, (b) the factor structure of PCS-YSR based on the unidimensional structure (Barber, 1996), combined and separately for mother and father forms, (c) gender invariance across girl and boy adolescents to assess the gender discrepancies in the perception of parental psychological control concept, (d) concurrent validity by the link of the mother and father psychological control with internalizing and externalizing behavior problems, as well as the discriminant validity, and (e) the correlation of the mother and father psychological control with children’s gender. We hypothesized that mothers’ and fathers’ psychological control would have a positive correlation with both internalizing and externalizing behavior problems in children.

## Materials and methods

### Research design

The current validation study was designed as a cross-sectional research (Kesmodel, 2018; Wang and Cheng, 2020).

### Participants

The sample was comprised of 1,453 Iranian high school students (49.2% boys; age range = 14–18 years; *M*_*age*_ = 15.48, *SD* = 0.97). The inclusion criteria were age between 14 and 18 years old and attending high school. Using convenience sampling method, the participants were recruited from 9th (16%), 10th (37.9%), 11th (27.6%), and 12th grades (18.6%) of only boy schools (*n* = 3) and only girl schools (*n* = 3) in the city of Tehran. Regarding fathers’ and mothers’ educational level, 21.8 and 18.5% had academic education, 74.7 and 77.4% had a diploma or lower education, and a mere of 3.5 and 4.1% had no formal education. Most fathers (95.7%) were employed, while only 15.5% of mothers were employed. Also, 3.7 and 84.4% of fathers and mothers were unemployed/housekeeper, and 0.6 and 0.1% of fathers and mothers were retired, respectively. Family structure included 89.9% adolescents living with both parents, 8.7% living with a single parent, and 1.4% living with others or alone.

### Materials/instruments

#### Parental Psychological Control

The 8-item self-administered measure of the Parental Psychological Control Scale-Youth Self-Report (PCS-YSR) was developed by (PCS-YSR; Barber, 1996) to assess parental psychological control. Adolescents rated how much items could correctly describe their parents in separate forms, where higher scores were reflective of higher level of psychological control. Items were rated on a Likert scale, ranging from 1 (*not like him/her*) to 3 (*a lot like him/her*). The sample item from the scale included: “*My mother/father is a person who always often interrupts me*”. Adequate internal consistency was reported in the study of Rodríguez-Menéndez et al. (2021), with *Cronbach*’s alphas of 0.83 and 0.76. for maternal and paternal psychological control in the Spanish population.

#### Adolescence Behavior Problems

Youth Self-Report (YSR; Achenbach, 1991) was used to measure behavior problems among adolescents. Internalizing scale comprised three subscales of: (a) anxious/depressed, (b) withdrawal/depressed and (c) somatic complaints. The externalizing scale included: (a) rule-breaking, and (b) aggressive behavior. On a Likert scale, the questions were rated between 0 (*not true*) and 2 (*very true or often true*). The Persian version of YSR (Fadaie et al., 2009) was used in this study and the alphas were 0.92 and 0.91 for Internalizing and Externalizing problems, respectively.

### Procedure

To translate the PCS-YSR, a bilingual team including a linguist and three experts in mental health translated the scale into the Persian language, and afterward, back-translated it into the English language based on the back-translation guidelines (Guillemin et al., 1993). The linguist expert checked the back translated version and established its consistency with the original scale. With conducting a pilot study, thirty high school students (50% girls) primarily completed the PCS-YSR to evaluate its reliability and validity, as well as to answer questions rated on a scale between 0 = “*not understandable*” to 5 = “*quite understandable*” regarding the Persian scale’s clarity. The evaluation showed 0.98% of participants in this pilot group found items intelligible, hence, the item revision was unnecessary. We did not include these students in the original study. Next, 16 schools from 16 diverse districts of Tehran city (four schools for each of lower city areas, lower middle areas, upper middle areas, and upper city areas) were invited to participate in this research. Among them, 6 schools from all districts (one school from lower city areas, two from lower middle areas, two from upper middle areas, and one from upper city areas) accepted the invitation and took part (response rate at the school level = 37.5%). A total of 1,800 students met our inclusion criteria. A majority of 1,453 out of 1,800 students accepted to take part (verbal assent to participate) and all of their parents signed the consent forms (the response rate at the student level = 85.28%). They were provided with a link for completing the online questionnaires. This study received approval from the ethics board of the Iran University of Medical Sciences (approval code = IR.IUMS.REC.1400.084), and is also in compliance with the Declaration of Helsinki developed by the World Medical Association (WMA, 2000) that specified the ethical principles when human subjects are involved (Bošnjak, 2001; Tyebkhan, 2003).

### Statistical analysis

To conduct the data screening, IBM SPSS Statistics (Version 28) was used. As indicated in Table 1, all items of PCS-YSR were homogeneous with no missing data, because the format of the response sheet in online data gathering needed to be submitted by the user after responding to all items (*n* = 1,453). All items met the univariate outlier criteria [−2.00 > *Z _*x*_* < +2.00]. The decision to keep or remove outliers was made based on the comparison of the original mean with a 5% trimmed mean. That is, we conducted data analyses using original data with keeping the outliers, along with using robust estimation for estimating related parameters (Tabachnick et al., 2007). We applied maximum likelihood with robust standard errors (MLR) estimation method, using Mplus version 8.8 (Asparouhov and Muthén, 2022), to test the *a priori* model of the confirmatory factor structure of the PCS-YSR. This type of analysis provides less bias and more accurate results for ordinal Likert-type scales (Mindrila, 2010; Li, 2016). As depicted in Table 1, the test of the assumption of normality revealed a mostly negative but non-substantial skewness in all items (Gravetter et al., 2020).

**TABLE 1:** Items mean, standard deviation, skewness, kurtosis, corrected item-total correlations, and reliability of PCS-YSR.

	Item No.	M	SD	SK	KU	r^cs^	Ordinal alpha	OACID	AVE	CR	Factor loadings	Total M (SD)	Boys M (SD)	Girls M (SD)
Maternal control	My Mother is a person who…								0.54	0.81		11.30 (2.68)	11.55 (2.68)	11.04 (2.57)
	1. … Is always trying to change how I feel …	1.677	0.489	−0.487	−1.098	0.23		0.92			0.26			
	2. … Changes the subject whenever I have …	1.327	0.478	0.862	−0.930	0.54		0.89			0.57			
	3. … Often interrupts me.	1.313	0.481	1.031	−0.378	0.57		0.88			0.62			
	4. … Blames me for other family …	1.310	0.476	1.001	−0.535	0.60	0.90	0.88			0.69			
	5. … Brings up past mistakes when …	1.466	0.520	0.372	−1.323	0.59		0.88			0.66			
	6. … Is less friendly with me if I do …	1.373	0.495	0.671	−1.187	0.63		0.88			0.72			
	7. … Will avoid looking at me when I have …	1.390	0.501	0.619	−1.202	0.61		0.88			0.64			
	8. … If I have hurt her feelings, stops talking …	1.444	0.521	0.488	−1.172	0.56		0.88			0.58			
Paternal control	My Father is a person who…								0.59	0.83		11.07 (2.67)	11.07 (2.68)	11.08 (2.67)
	1. … Is always trying to change how I feel …	1.625	0.509	−0.221	−1.264	0.39	0.92	0.93			0.42			
	2. … Changes the subject whenever I have …	1.257	0.446	1.247	−0.073	0.55		0.91			0.58			
	3. … Often interrupts me.	1.258	0.450	1.294	0.189	0.61		0.90			0.65			
	4. … Blames me for other family …	1.296	0.465	1.030	−0.578	0.62		0.90			0.71			
	5. … Brings up past mistakes when …	1.473	0.519	0.330	−1.376	0.59		0.91			0.67			
	6. … Is less friendly with me if I do …	1.348	0.489	0.805	−0.936	0.65		0.90			0.73			
	7. … Will avoid looking at me when I have …	1.368	0.493	0.692	−1.155	0.61		0.91			0.63			
	8. … If I have hurt her feelings, stops talking …	1.452	0.514	0.381	−1.412	0.57		0.91			0.58			

M, mean; SD, standard deviation; SK, skewness; KU, kurtosis, r^cs^, corrected item-total correlation; OACID, ordinal alpha coefficient if item deleted; AVE, average variance extracted (AVE) for discriminant validity; CR, composite reliability.

Data analyses was as follows: First, as recommended for ordinal Likert-type scales, the internal consistency was examined using the composite reliability and ordinal alpha reliability coefficients by semTools and psych Packages (Revelle, 2015) in R version 4.1.2 (Revelle, 2017). Ordinal alpha is the equivalent of *Cronbach*’s alpha coefficient, which instead of the Pearson correlation matrix, are according to the polychoric correlation matrix (Zumbo et al., 2007; Gadermann et al., 2012). As suggested by Cicchetti (1994), a correlation coefficient of 0.70 or higher was considered as an acceptable level of internal consistency of the items. The corrected item-total correlation’s values were interpreted as: 0–0.19 (not well discrimination), 0.2–0.39 (good discrimination), and 0.4 and above (very good discrimination) (Ferketich, 1991). The interpretation of mean of inter-item correlations was classified as: poor (not correlated well; below 0.15), and good (0.15–0.50). In addition, values of Inter-item correlation higher than 0.50 are the indicator of strong correlation between items, which may show that items have repetitive content (Hair et al., 2006).

Second, to test the construct validity of the PCS-YSR, the CFA was conducted using four models. Model 1 (M_1_) examined a general factor for mothers resembling the exploratory factor analysis conducted by (Barber, 1996), in which the total 8 items loaded on one common factor of parental psychological control to test the unidimensional model of assumed latent factor and include random measurement error and indicator-specific variance (Gustafsson and Åberg-Bengtsson, 2010). Model 2 (M_2_) consisted of the same model but for fathers. Model 3 (M_3_) examined a unidimensional-factor with correlated errors model for mothers, and Model 4 (M_4_) tested the same as model 3, but for fathers.

To assess the “goodness-of-fit,” the following statistical tests and indices were employed (acceptable values in parenthesis): the chi-square (*χ*^2^; desirable that *p* > 0.05), the standardized root mean square residual (SRMR < 0.05), the root mean square error of approximation (RMSEA < 0.05), and its 90% confidence interval, the comparative fit index (CFI > 0.95), the Tucker–Lewis index (TLI > 0.95), and the normalized chi-square (*χ*^2^/df < 3) (Bentler and Bonett, 1980; MacCallum et al., 1996; Maruyama, 1997; Loehlin, 2004; Miles and Shevlin, 2007). The exact fit is defensible when *χ^2^* is not significant, regardless of the SRMR value. In case chi-square is significant, SRMR ≤ 0.08, and standard residuals are all small (| r_*res*_| < 0.1), approximate fit is tenable. Finally, if chi-square is significant and SRMR > 0.08, poor fit is concluded. To further compare the competing models’ fit, the Bayesian Information Criterion (BIC) was also reported. The model that possesses the lower BIC value fits best.

Third, after model selection, measurement invariance across genders was tested. Invariance of factorial structure/pattern, factor loadings (weak invariance), item intercepts (strong), and finally item residuals or unique variances (strict) were examined. In the case of invariance, first, we compared the RMSEA values and RMSEA confidence intervals of the nested models. For instance, when comparing the configural and metric invariance models, falling the RMSEA values within the confidence intervals of one another is an indicative of metric invariance. Then, we tested to observe any changes in the nested models’ CFI, RMSEA, and SRMR. Measurement invariance can be supported if two of the following indices are satisfied: ΔCFI ≤ 0.01, ΔSRMR ≤ 0.01 and ΔRMSEA ≤ 0.015 for the test of intercept invariance and residual invariance, and ΔCFI ≤ 0.01, ΔSRMR ≤ 0.03, and ΔRMSEA ≤ 0.015 for the test of factor loading invariances (Cheung and Rensvold, 1999, 2002; Sass et al., 2014).

Fourth, concurrent validity was evaluated by the *point-biserial* correlation and *Kendall’s coefficient* of rank correlation (*τ_*b*_*) of maternal and paternal psychological control with behavior problems, gender, and grade because the data showed evidence of non-normality. The interpretation of correlation coefficients was based on Cohen (1988) suggestion: the effect sizes of small = ≤0.10, medium = 0.30, large = 0.50, and very large = ≥ 0.70. Discriminant validity was also conducted through Average Variance Extracted (AVE).

## Results

### Internal reliability

Table 1 presents the descriptive statistics of PCS-YSR. Almost all the items had a moderate positive relationship with each other based on the corrected item-total correlation for maternal and paternal forms’ items —with values ranging from 0.23 to 0.63 (mothers’ form) and 0.39 to 0.65 (fathers’ form). The means of inter-item correlation, ordinal alpha reliability, *Cronbach*’s alphas, and composite reliability were 0.36, 0.90, 0.81, and 0.81 for maternal control and 0.40, 0.92, 0.83, and 0.83 for paternal control, respectively.

### Factor structure

As indicated in Table 2, unidimensional model (M_1_ and M_3_) did not meet most of the specified fit criteria (i.e., CFI > 0.95, RMSEA < 0.05, TLI > 0.95, *χ^2^*/df < 3). As part of the next step, after freeing the error covariance of 2 pairs of items, a one-factor item-correlated errors model for mothers (M_2_; *χ^2^*/df = 3.54; CFI = 0.98; TLI = 0.97; and RMSEA = 0.04; 90% CI = 0.03 to 0.05) and fathers (M_4_; *χ^2^*/df = 3.34; CFI = 0.98; TLI = 0.97; and RMSEA = 0.04; 90% CI = 0.02 to 0.05) provided a better fit.

**TABLE 2:** Invariance analysis for CFA of the PCS-YSR across gender in one-factor oblique correlated errors model.

Model	χ^2^(df)	χ^2^/df	AIC	BIC	CFI	TLI	RMSEA	SRMR	Base model	Δχ^2^	ΔCFI	Δ TLI	Δ RMSEA
M_1_	219.726 (20)	10.98	14316.006	14444.030	0.923	0.892	0.081 (0.070–0.090)	0.041	**–**	**–**			
M_2_	63.641 (18)	3.54	14112.778	14251.470	0.982	0.973	0.041 (0.030–0.052)	0.024	M_1_	130.43***	0.059	0.081	0.04
M_3_	234.135 (20)	11.71	13477.171	13605.195	0.923	0.893	0.084 (0.074–0.093)	0.041	M_1_	−14.40	0.000	0.001	0.003
M_4_	60.256 (18)	3.34	13236.043	13374.735	0.985	0.976	0.039 (0.029–0.050)	0.021	M_3_	51.91***	0.062	0.083	0.045
**Measurement invariance for mothers across child gender**													
Boys	40.336 (18)	2.24	7201.987	7322.624	0.983	0.973	0.04 (0.024–0.057)	0.04	M_2_	19.92	0.001	0.000	0.001
Girls	41.823 (18)	2.32	6872.420	6993.124	0.981	0.971	0.042 (0.025–0.058)	0.026	M_2_	21.82	0.001	0.002	0.001
Configural	82.181 (36)	2.28	14074.407	14351.792	0.982	0.972	0.041 (0.029–0.053)	0.027	M_2_	19.47	0.000	0.001	0.000
Metric	91.062 (43)	2.11	14068.643	14308.688	0.982	0.977	0.038 (0.027–0.049)	0.029	Configural	7.67	0.000	0.005	0.003
Scalar	91.062 (43)	2.11	14068.643	14308.688	0.981	0.976	0.038 (0.027–0.049)	0.032	Metric	0.000	0.001	0.001	0.000
**Measurement invariance for fathers across child gender**													
Boys	42.943 (18)	2.38	6699.132	6819.769	0.982	0.973	0.043 (0.026–0.059)	0.027	M_4_	17.31	0.003	0.003	0.004
Girls	42.058 (18)	2.38	6564.559	6685.263	0.983	0.973	0.042 (0.025–0.058)	0.025	M_4_	18.20	0.002	0.003	0.003
Configural	84.991 (36)	2.36	13263.691	13541.076	0.983	0.973	0.042 (0.031–0.054)	0.026	M_4_	24.35	0.002	0.003	0.003
Metric	92.397 (43)	2.14	13254.317	13494.362	0.983	0.977	0.045 (0.042–0.048)	0.029	Configural	4.73	0.000	0.004	0.003
Scalar	92.397 (43)	2.14	13254.317	13494.362	0.982	0.977	0.039 (0.028–0.050)	0.029	Metric	0.000	0.001	0.000	0.006

M_1_ = one-factor model for mothers, M_2_ = one-factor and correlated errors model for mothers (items: 7–8, and 2–3), M_3_ = one-factor model for fathers, M_4_ = one-factor and correlated errors model for fathers (items: 7–8, and 2–3), χ^2^= chi-square, df, degrees of freedom; TLI, tucker–Lewis index; CFI, comparative fit index; χ^2^/df = normal chi-square; AIC, Akaike’s information criterion; BIC, Bayesian information criterion; SRMR = standardized root mean square residual; RMSEA, root mean square error of approximation; Δχ^2^= significant χ^2^ change indicates non-invariance of the model that hierarchically was compared with the previously ordered model. ***p < 0.001.

### Model selection

**FIGURE 1:**
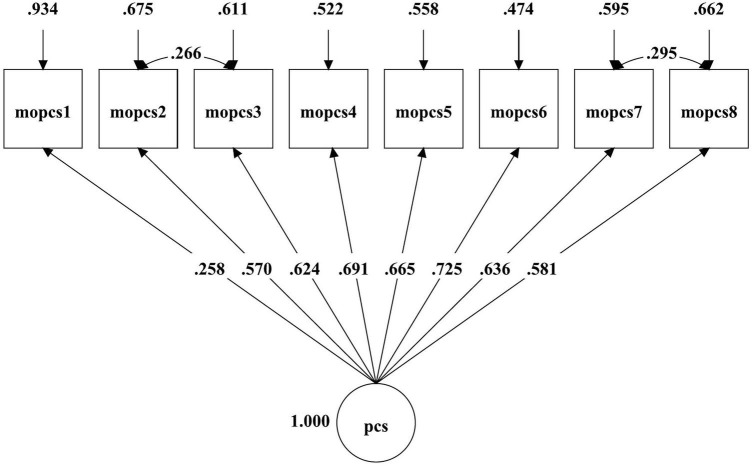
The one-factor correlated errors model of maternal psychological control.

**FIGURE 2:**
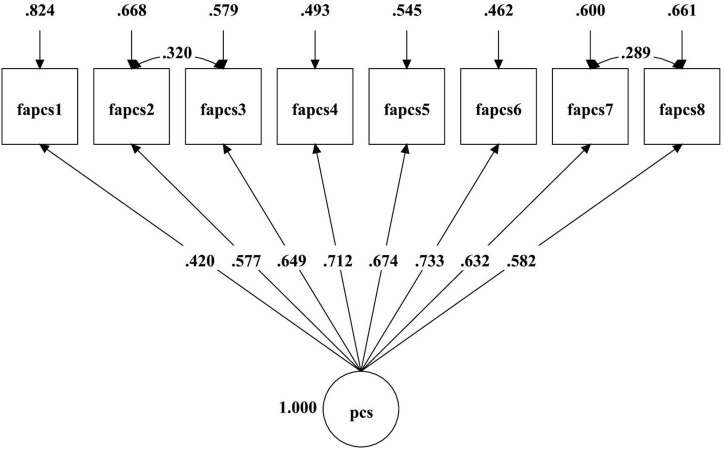
The one-factor correlated errors model of paternal psychological control.

The principle of parsimony (Bollen, 1989), results in Figures 1, 2 and Table 2 showed the one-factor correlated errors model (M_2_) for mothers and (M_4_) for fathers fits the data well, with those of corresponding models as the baseline/null model, then indicated that the one-factor correlated errors model (M_2_ and M_4_) were the optimal/parsimonious models for both parents.

### Measurement invariance across gender

In seeking whether measurement of PCS-YSR was equivalent across the gender of the child (n _*girls*_ = 769 and n _*boys*_ = 765) for mothers’ and fathers’ forms, we ran the multi-group CFA. Primarily, we ran the CFA in a total sample and then, separately for both girls and boys in order to achieve an adequate fitness for each baseline model, on the basis of the parsimony and meaningfulness perspective (Werts et al., 1976). As seen in Table 2, the one-factor model with correlated error covariance was run (diagonal error covariance which freed between items including 8–7, and 2–3) in both boys’ and girls’ groups, separately for mothers’ and fathers’ forms, to get in a baseline model (Werts et al., 1976). Configural invariance or factorial structure/pattern, strong factorial invariance, and weak factorial invariance were evaluated (Byrne et al., 1989; Meredith, 1993; Cheung and Rensvold, 2002).

As indicated in Table 2, the hypothesized measurement invariances of PCS-YSR for both parents (i.e., unidimensional and correlated errors model) fitted the data well, showing that the same construct was being measured across gender of the child. In other words, the equivalent factor structure, pattern of factor-indicator relationships, factor loadings, and item intercepts were found across the child’s gender in both parents’ forms. Finally, according to Table 2, the comparison of configural with baseline model, metric with configural model, scalar with metric model, strict with scalar model were conducted. It could be concluded that the one-factor model with correlated errors is the parsimonious model across gender of child for both parents’ forms.

### Concurrent and discriminant validity

Table 3 presents the correlation coefficients between the maternal and paternal control; based on the unidimensional model. Maternal and paternal control had a significant association with each other, with correlation coefficients of 0.62 and 0.64 for girl and boy adolescents, respectively. As displayed in Table 3, the maternal and paternal psychological control had a significant positive correlation with YSR (*p* < 0.01), showing its concurrent validity. The correlation coefficients for internalizing problems were 0.44 (with maternal control) and 0.47 (with paternal control), and for externalizing problems were 0.43 (with maternal control) and 0.44 (with paternal control). The relations between demographic variables (gender and grade) and PCS-YSR were examined by *Kendall*’s coefficient of rank correlation (*τ_*b*_*). As Table 3 depicts, no significant relations were found between grade of children with both mother and father forms of psychological control. Adolescents’ gender was significantly and negatively correlated with maternal (*r* = −0.09, *p* < 0.01), but not paternal psychological control. Thus, mothers have a more likelihood to show psychological control toward their sons than daughters. However, paternal psychological control remains the same, regardless of the child’s gender. Discriminant validity through AVE (Table 2; Factor Analysis) were also acceptable—-0.54 and 0.59 for maternal and paternal psychological control, respectively.

**TABLE 3:** Correlations between the PCS-YSR, gender, grade, and behavior problems (*n* = 1,453).

Construct	1	2
1. Maternal psychological control	1	0.62**
2. Paternal psychological control	0.64**	1
3. Gender	−0.09**	0.01
4. Grade	0.04	0.03
5. Anxious/depressed	0.40**	0.43**
6. Withdrawal/depressed	0.37**	0.40**
7. Somatic complaints	0.41**	0.44**
8. Internalizing problems	0.44**	0.47**
9. Rule-breaking behavior	0.37**	0.35**
10. Aggressive behavior	0.41**	0.46**
11. Externalizing problems	0.43**	0.44**

The inter-correlation related to Parental Psychological control below the diagonal is for girl adolescents, and above the diagonal is for boy adolescents. **p < 0.01.

## Discussion

The current study sought to investigate the psychometric characteristics of the adapted version of the PCS-YSR scale among Iranian adolescents. Primarily, the result supported the reliability of this scale. A unidimensional model fits the data best, that was quite consistent with the original model of Barber (1996). Further support was also provided for the concurrent validity of the PCS-YSR scale, with demonstrating a positive correlation with internalizing and externalizing problems. In addition, the result supported the gender invariance of this scale, while mothers manifested higher level of psychological control toward their sons. Overall, the current results confirmed that PCS-YSR is a reliable and valid scale to be applied in the Iranian adolescent population.

Regarding the scale’s reliability, the *Cronbach*’s alpha, Ordinal alpha, and composite reliability coefficients were satisfactory for mother and father psychological control. The means of inter-item correlations were sufficient (0.36 for maternal control and 0.40 for paternal control), since poor correlation shows a lack of similarity between items, and strong correlation implies that items are quite similar and are measuring the exact same content (Hair et al., 2006). The values of corrected item-total correlation for maternal and paternal forms’ items have shown good and very good reliability (Ferketich, 1991). These findings were consistent with earlier evidence (e.g., Rodríguez-Menéndez et al., 2021) and generally supported the excellent reliability of PCS-YSR among Iranian adolescents.

CFA findings revealed that the structure of PCS-YSR with one factor could describe the data best. Both paternal and maternal psychological control CFA findings revealed a good equivalence with the original one-factor version in our sample. It suggests that the structure of PCS-YSR is consistent among mothers and fathers. This result is in line with the single factor initially developed by Barber (1996) and further studies (Galambos et al., 2004; Snoek et al., 2007; Li et al., 2013). Moreover, the highest factor loadings was found in items 4 to 6, which mesh with the results of Barber et al. (2012) and Rodríguez-Menéndez et al. (2021). In general, the optimal fit indices were found for both each item and the overall measure in the present study.

The multi-group CFA revealed equalities in the item-to-item correlation matrices, as well as configure, metric, scalar, and strict invariance (Vandenberg and Lance, 2000; Byrne, 2001; Cheung and Rensvold, 2002; Meredith and Teresi, 2006) across gender. The measurement invariance indicates that girls and boys perceived the scale items equally and the similar construct was measured across gender. This finding implies that any discrepancies between girls and boys in the level of parental psychological control would be a result of real differences in the latent factor, instead of structural differences (variation in the perception of the items’ concepts) (Vandenberg and Lance, 2000).

The concurrent validity of PCS-YSR was supported through its moderate positive association with behavior problems. Unsurprisingly, the psychological control scores in both mothers and fathers were positively correlated with internalizing problems, including anxious-depressed, depressed-withdrawal, and somatization symptoms. It was expected because internalizing problems are believed to be the underlying developmental and psychological outcome of parental psychological control (Barber, 1996). The critical tone, which usually accompanies parental psychological control, may insecure adolescents regarding their capacities and suppress their sense of competence. Furthermore, perceived parental psychological control may weaken the parent-adolescent bonding-a threat that may be transferred to other relationships (Barber et al., 2005b). Consistent with earlier findings demonstrating that manipulative parenting strategies employed in parental psychology control may elevate susceptibility to internalizing problems (Wouters et al., 2018; Gorostiaga et al., 2019; Cai and Tu, 2020). Moreover, paternal and maternal psychological control had a positive correlation with externalization problems of rule-breaking and aggressive behavior. Intruding behaviors, personal attack, and stifling autonomy in the context of psychological control may elevate insecurity and frustration (De Kemp et al., 2006) and prevent children from fostering self-regulation, which result in deviant and impulsive behavior, or the willing to take risks and break social norms (Yang et al., 2022). This finding is also consistent with several previous evidence (e.g., Pace et al., 2018; León-del-Barco et al., 2019; Yan et al., 2021).

As for the role of gender on the level of psychological control, we found fathers treated their daughters and sons with similar levels of psychological control. Mothers, on the contrary, appeared to employ higher level of psychological control strategies toward their sons, than their daughters. This result was in line with prior evidence (De Kemp et al., 2006; Endendijk et al., 2016) and provided partial support for the bio-social theory of gender differences (Wood and Eagly, 2002, 2012). Based on this theory, members of the society are exposed to social norms and expectations associated with gender. Parents, peers, and the broader society provide models and cues for what is considered appropriate behavior for boys and girls and how they should be treated differently. In the context of parenting, mothers are encouraged to treat their girls with more gentle and sensitive parenting strategies (Tamis-LeMonda et al., 2009; Mandara et al., 2012), while act with power and assertiveness toward their sons (Kochanska et al., 2009; Tamis-LeMonda et al., 2009). Another underlying factor in this differential treatment might be the bidirectional nature of parental psychological control-child behavior link. Indeed, psychological control may conceivably be driven by child behavior (Larsson et al., 2008). On the other hand, the bio-social theory recognizes that biological factors, such as hormonal differences (e.g., higher levels of testosterone in boys) can contribute to some gender-specific behaviors (including aggression and risk-taking) (Wood and Eagly, 2002, 2012). With this presupposition, one may argue that as boys show higher rate of aggressive and disruptive behavior (Loeber et al., 2013), they have more likelihood to face with higher level of parental psychological control by their mothers-as their first caregiver. However, the gender difference in this study was negligible; thus, for justifying or speculating the probable mechanisms that explain this distinct pattern, the acquisition of further empirical evidence is needed to test the stability and reliability of our results in other cultural backgrounds and to explore the underlying mechanisms driving gender differences in psychological control.

The acceptable discriminant validity of the PCS-YSR was supported through finding the recommended score of AVE (<0.5) for both maternal and paternal forms. This result implies that the variance captured by the construct exceeds the variance due to measurement error, supporting the discriminant validity of the construct and individual indicators (Fornell and Larcker, 1981; Henseler et al., 2015). AVE is also a rigorous test for convergent validity, when comparing it with composite reliability. In case of only taking composite reliability into account (even when more than 50% of variance is explained by error), convergent validity can be considered as sufficient (Voorhees et al., 2016; Kock, 2019).

### Limitation, future directions, and implications

Our findings should be interpreted considering some limitations, which can point to promising areas for further investigation. First, we used the self-report measures to collect the present data; specifically, the PCS-YSR only captures the adolescent perception of the parental psychological control. Although measuring this perception is necessary due to its effect on internalizing behavior problems (Brenning et al., 2015; van der Kaap-Deeder et al., 2017), it may also include bias and limit the data’s accuracy. Future studies should simultaneously use a multiple-informant approach (including parents’, teachers’, and peers’ reports) or other measurement tools, such as interviews and real-life observation for assessing parental psychological control. These tools may capture a more comprehensive picture of the parenting practices of Iranian parents. Second, it should be considered that the current study is cross-sectional, so it cannot convey the PCS-YSR’s predictive validity. Future studies may be more beneficial if their analysis also focuses on longitudinal studies to examine the bidirectional or reciprocal relationship between parental psychological control and behavior problems during time. Third, our study used a version of the scale that measures the overall parental psychological control, while two types of control, the achievement-oriented and the dependency-oriented psychological control are recently proposed (Soenens et al., 2010). Achievement-oriented psychological control is attributed to the parents’ attempt to impose high standards for successful function to their children. Dependency-oriented psychological control is related to the strategies for maintaining the physically and emotionally close relationship with children. Parents might employ one of these types of psychological control based on their characteristics, such as high perfectionism and not having boundaries with other family members (Soenens et al., 2010). Due to their distinctive orientations, these two dimensions of control target different maladjustments. Since dependency-oriented psychological control inevitably stifles the child’s independent orientation toward parents, it may exacerbate internalizing problems (Soenens et al., 2012; Gargurevich and Soenens, 2016). On the other hand, achievement-oriented control makes children prone to maladaptive perfectionism due to the pressure they experience to behave ideally (Clark and Coker, 2009). Thus, utilizing this person-oriented approach is important for the future research to specify the parental psychological control’s orientation toward achievement or dependency (Soenens et al., 2010), particularly because of their distinctive effect on adolescent’s maladjustment.

The present study has three main implications for research and practice. Generally, our results demonstrate that the PCS-YSR is a reliable index of the degree of parental psychological control and possesses capacity to easily screen the adolescents who are psychologically controlled by their parents in clinical context. Specifically, our results provide a wide range of information for family therapists, social workers, teachers, and adolescent psychologists in the Iranian culture. For example, it is documented that parental psychological control is in fact a destructive parenting behavior, linking to youth’s behavior problems, that needs to be identified and targeted in child psychology. Another vital finding was the significant impact of gender roles on parental control in Iranian culture, suggesting that such gender differences may be found across cultures, and should be taken into account when targeting parental psychological control by interventions in various contexts.

## Conclusion

PCY-YSR is an internationally well-stablished scale to measure the negative aspect of parental control-psychological control. The present findings supported the construct and concurrent validity, reliability, and the gender invariance of the Persian version of PCY-YSR among an adolescent population. Findings of construct validity confirmed the original unidimensional model of the PCY-YSR scale for both mother and father control. Concurrent validity was established through the relationship of PCS-YSR to internalizing and externalizing behavior problem. PCS-YSR proved to be reliable in the current sample and invariant across two dyads of father-son and father-daughter, while different across two dyads of mother-son and mother-daughter. As a concluding remark, the PCY-YSR possesses the psychometric soundness for evaluating perceived parental psychological control among adolescents in Iran.

## Data availability statement

The raw data supporting the conclusions of this article can be provided by the corresponding author upon reasonable request.

## Ethics statement

The studies involving human participants were reviewed and approved by the Iran University of Medical Sciences (approval code = IR.IUMS.REC.1400.084). Written informed consent to participate in this study was provided by the participants’ legal guardian/next of kin.

## Author contributions

PS and MH: conceptualization, design, methodology, data collection, investigation, and project administration. BI and MH: formal analysis. MH: supervision. PS, SM, and MH: writing the original draft. PS, ET-C, and MH: revising the draft. All authors have contributed to the conception and design of the study, drafted or revised this manuscript, reviewed the final version of this manuscript before submission, and agreed to be accountable for all aspects of the work.
