# Corrigendum: Confirmatory factor analysis and gender invariance of the Persian version of psychological control scale: association with internalizing and externalizing behavior problems

**DOI:** 10.3389/fpsyg.2024.1373512

**Published:** 2024-02-02

**Authors:** Mojtaba Habibi Asgarabad, Pardis Salehi Yegaei, Sima Mokhtari, Balal Izalnoo, Elizabeth Trejos-Castillo

**Affiliations:** ^1^Health Promotion Research Center, Iran University of Medical Sciences, Tehran, Iran; ^2^Department of Psychology, Norwegian University of Science and Technology, Trondheim, Norway; ^3^Department of Health Psychology, School of Behavioral Sciences and Mental Health (Tehran Psychiatric Institute), Iran University of Medical Sciences, Tehran, Iran; ^4^Positive Youth Development Lab, Human Development and Family Sciences, Texas Tech University, Lubbock, TX, United States; ^5^Center of Excellence in Cognitive Neuropsychology, Institute for Cognitive and Brain Sciences, Shahid Beheshti University, Tehran, Iran; ^6^Department of Clinical Psychology, School of Behavioral Sciences and Mental Health (Tehran Psychiatric Institute), Iran University of Medical Sciences, Tehran, Iran; ^7^Faculty of Psychology, Kharazmi University, Tehran, Iran; ^8^Human Development and Family Sciences, Texas Tech University, Lubbock, TX, United States

**Keywords:** internalizing and externalizing behavior problems, gender invariance, parental psychological control, reliability, validity

In the published article, there was an error in the correspondence details. As well as Pardis Salehi Yegaei, Mojtaba Habibi Asgarabad should also be listed as a corresponding author. The complete correspondence details are shown below:

^*^ **Correspondence:**

Pardis Salehi Yegaei

Pardis_salehi2012@yahoo.com

Mojtaba Habibi Asgarabad

Mojtaba.h.asgarabad@ntnu.no

The authors apologize for this error and state that this does not change the scientific conclusions of the article in any way. The original article has been updated.

